# Increasing spillover of highly pathogenic avian influenza A (H5N1) virus to mammals

**DOI:** 10.1016/j.nmni.2024.101459

**Published:** 2024-08-19

**Authors:** Shao-Lun Zhai, Sheng-Nan Chen, Jieshi Yu, Bo Liu, Handuo Jia

**Affiliations:** Institute of Animal Health, Guangdong Academy of Agricultural Sciences, Key Laboratory of Livestock Disease Prevention of Guangdong Province, Scientific Observation and Experiment Station of Veterinary Drugs and Diagnostic Techniques of Guangdong Province, Ministry of Agriculture and Rural Affairs, Key Laboratory for Prevention and Control of Avian Influenza and Other Major Poultry Diseases, Guangzhou, 510640, China; Guangzhou Sino-science Gene Testing Service Co., Ltd, Guangzhou, 510700, China; Agro-Biological Gene Research Center, Guangdong Academy of Agricultural Sciences, Guangzhou, 510640, China; Institute of Animal Health, Guangdong Academy of Agricultural Sciences, Key Laboratory of Livestock Disease Prevention of Guangdong Province, Scientific Observation and Experiment Station of Veterinary Drugs and Diagnostic Techniques of Guangdong Province, Ministry of Agriculture and Rural Affairs, Key Laboratory for Prevention and Control of Avian Influenza and Other Major Poultry Diseases, Guangzhou, 510640, China; College of Animal Science and Technology, Zhongkai University of Agriculture and Engineering, Guangzhou, 510000, China; Institute of Animal Health, Guangdong Academy of Agricultural Sciences, Key Laboratory of Livestock Disease Prevention of Guangdong Province, Scientific Observation and Experiment Station of Veterinary Drugs and Diagnostic Techniques of Guangdong Province, Ministry of Agriculture and Rural Affairs, Key Laboratory for Prevention and Control of Avian Influenza and Other Major Poultry Diseases, Guangzhou, 510640, China; College of Veterinary Medicine, Xinjiang Agricultural University, Urumqi, 830052, China

**Keywords:** Highly pathogenic avian influenza, H5N1 virus, Spillover, Mammals, Cross-species transmission

## Abstract

• 1.Increasing spillover of highly pathogenic avian influenza A (H5N1) virus to sea mammals.
• 2.Increasing spillover of highly pathogenic avian influenza A (H5N1) virus to fur mammals.
• 3.Increasing spillover of highly pathogenic avian influenza A (H5N1) virus to ruminant animals.
• 4.Cross-species transmission of highly pathogenic avian influenza A (H5N1) virus between ruminant animals and humans.
• 5.Highly pathogenic avian influenza A (H5N1) virus RNA was present in milk.

Influenza virus (IV) is a negative-sense, single stranded segmented RNA virus, and belongs to the family *Orthomyxoviridae*. IV contains four genera, designated as *Alphainfluenzavirus*, *Betainfluenzavirus*, *Gammainfluenzavirus* and *Deltainfluenzavirus*. Among them, only one type of species is known, and these are named Influenza A virus (IAV), Influenza B virus (IBV), Influenza C virus (ICV), and Influenza D virus (IDV). According to the antigenic variations of the surface glycoproteins, hemagglutinin (HA), neuraminidase (NA) or hemagglutinin-esterase fusion (HEF) protein, IVs are divided into distinct subtypes or lineages. In particular, IAVs are very diverse, containing 18 HA (H1-H18) subtypes and 11 NA (N1-N11) subtypes [1].

IAV mainly infects over than 100 species of birds and a wide range of mammals (including humans), bringing huge lesions and deaths for the hosts. IBV mainly infects humans, pigs, and seals, and causes the greatest lesions to humans. ICV mainly infects humans and pigs. IDV mainly infects cattle, pigs, camels, horses, sheep, and goats. Compared to the first two IVs, the latter two IVs have less lesions to the host [1].

In recent years, IVs, especially IAVs, are expanding their host. Highly pathogenic avian influenza A (HPAI H5N1) virus, a subtype of IAVs, was reported in mammals (including skunks, foxes, bears, minks, seals, porpoises, sea lions, and dolphins, etc.) from Asia, America, and Europe [2]. Since the March of 2024, in the United States, HPAI H5N1 viruses (clade 2.3.4.4b) began to jump into cows originating from Texas, Kansas, New Mexico, Idaho, and Michigan. Although cows routinely contract influenza viruses, this is the first time that a HPAI H5N1 virus has been found in cattle. According to the information from United States Department of Agriculture (USDA), about 10 % of affected herds had become ill. Sick cows had a mild illness, and produced less milk. Moreover, in early March of 2024, HPAI virus was also found in a goat in Minnesota, the first case in U.S. livestock. Preliminary investigation into the source of these infection suggests that it was caused by infected wild birds or humans by HPAI virus on farms or the farm surroundings [3].

Generally speaking, cattle were largely unaffected by IAVs, even though most of the domesticated and wild animals at the human-animal interface succumbed to infection over the past few decades [4]. Incredibly, HPAI H5N1 virus begin to cause the infection and/or death for its new uncommon host, especially sea mammals and ruminants, suggesting that the virus is adapting to mammalian infections [5]. Under the ongoing global outbreaks of HPAI H5N1 virus, it is very necessary to undergo vaccine immunization and viral surveillance in susceptible or possible susceptible animals.

## CRediT authorship contribution statement

**Shao-Lun Zhai:** Writing – review & editing, Writing – original draft, Supervision, Project administration, Funding acquisition, Data curation. **Sheng-Nan Chen:** Writing – review & editing, Writing – original draft, Data curation. **Jieshi Yu:** Writing – review & editing, Formal analysis, Conceptualization. **Bo Liu:** Writing – review & editing. **Handuo Jia:** Writing – review & editing.

## Declaration of competing interest

The authors declare that they have no known competing financial interests or personal relationships that could have appeared to influence the work reported in this paper.
